# Risk Factors Associated With SARS-CoV-2 Breakthrough Infections in Fully mRNA-Vaccinated Individuals: Retrospective Analysis

**DOI:** 10.2196/35311

**Published:** 2022-05-24

**Authors:** Cong Liu, Junghwan Lee, Casey Ta, Ali Soroush, James R Rogers, Jae Hyun Kim, Karthik Natarajan, Jason Zucker, Yehoshua Perl, Chunhua Weng

**Affiliations:** 1 Department of Biomedical Informatics Columbia University Irving Medical Center New York, NY United States; 2 School of Pharmacy Jeonbuk National University Jeonju Republic of Korea; 3 Department of Medicine Columbia University Irving Medical Center New York, NY United States; 4 Department of Computer Science New Jersey Institute of Technology Newark, NJ United States

**Keywords:** COVID-19, medical informatics, real-word evidence, vaccination, electronic health records

## Abstract

**Background:**

COVID-19 messenger RNA (mRNA) vaccines have demonstrated efficacy and effectiveness in preventing symptomatic COVID-19, while being relatively safe in trial studies. However, vaccine breakthrough infections have been reported.

**Objective:**

This study aims to identify risk factors associated with COVID-19 breakthrough infections among fully mRNA-vaccinated individuals.

**Methods:**

We conducted a series of observational retrospective analyses using the electronic health records (EHRs) of the Columbia University Irving Medical Center/New York Presbyterian (CUIMC/NYP) up to September 21, 2021. New York City (NYC) adult residences with at least 1 polymerase chain reaction (PCR) record were included in this analysis. Poisson regression was performed to assess the association between the breakthrough infection rate in vaccinated individuals and multiple risk factors—including vaccine brand, demographics, and underlying conditions—while adjusting for calendar month, prior number of visits, and observational days in the EHR.

**Results:**

The overall estimated breakthrough infection rate was 0.16 (95% CI 0.14-0.18). Individuals who were vaccinated with Pfizer/BNT162b2 (incidence rate ratio [IRR] against Moderna/mRNA-1273=1.66, 95% CI 1.17-2.35) were male (IRR against female=1.47, 95% CI 1.11-1.94) and had compromised immune systems (IRR=1.48, 95% CI 1.09-2.00) were at the highest risk for breakthrough infections. Among all underlying conditions, those with primary immunodeficiency, a history of organ transplant, an active tumor, use of immunosuppressant medications, or Alzheimer disease were at the highest risk.

**Conclusions:**

Although we found both mRNA vaccines were effective, Moderna/mRNA-1273 had a lower incidence rate of breakthrough infections. Immunocompromised and male individuals were among the highest risk groups experiencing breakthrough infections. Given the rapidly changing nature of the SARS-CoV-2 pandemic, continued monitoring and a generalizable analysis pipeline are warranted to inform quick updates on vaccine effectiveness in real time.

## Introduction

The ongoing global COVID-19 pandemic has infected hundreds of millions of people worldwide, imposing a tremendous burden on the global health care system. COVID-19 vaccines are currently the best defense against the rapidly evolving SARS-CoV-2, having demonstrated efficacy in preventing symptomatic COVID-19, while being relatively safe in trial studies [1-3]. In addition to the clinical trial studies, multiple studies have been conducted to confirm vaccine effectiveness using real-world observational data as well [4-9]. As of March 2022, over 200 million individuals in the United States had been fully vaccinated [10].

The Centers for Disease Control and Prevention (CDC) has reported vaccine breakthrough infections, defined as a fully vaccinated person getting infected with COVID-19 [11]. SARS-CoV-2 reinfection and vaccine breakthrough have now been frequently reported [12-17]. Newer variants of concern that now account for the majority of infections worldwide, including delta (B.1.617.2) and omicron (B.1.1.529), have also increased transmissibility and increased rates of vaccine breakthrough compared to older variants [18,19]. Given the concerns about vaccine breakthrough infections [20], studies have been conducted to confirm vaccine breakthrough infections with SARS-CoV-2 variants using genome sequencing [16] and to investigate clinical characteristics of the vaccine breakthrough infections [21-23]. Early reports have found breakthrough infections more often occur in individuals with solid organ transplants [24-27], obesity [28], hypertension [29], diabetes [29,30], congestive heart failure [29,31], chronic kidney disease (CKD) [22,32], lung diseases [33], dementia [34], and cancer [22,35-37]. Here, we retrospectively analyzed electronic health records (EHRs) from the Columbia University Irving Medical Center/New York Presbyterian (CUIMC/NYP) up to September 21, 2021, to systematically identify risk factors associated with breakthrough infections among fully messenger RNA (mRNA)–vaccinated individuals.

## Methods

### Ethical Considerations

The study adhered to the principles set out in the Declaration of Helsinki, with informed consent obtained from all participants. The Columbia University Health Sciences Institutional Review Board (IRB) reviewed and approved the study (IRB AAAR3954). The analysis in this study was conducted on the deidentified data.

### Study Design and Population

We used EHR data obtained from the NYP/CUIMC data warehouse. The NYP/CUIMC is a quaternary care academic medical center that includes an academic hospital, a children’s hospital, and a community-based hospital serving a diverse patient population in northern Manhattan, New York City (NYC). EHR data were collected and stored in the data warehouse during routine clinical care at the CUIMC/NYP. The EHR data were converted to the Observational Medical Outcomes Partnership (OMOP) common data model (CDM) version 5.0 [38]. All data involved in this analysis were collected up to September 21, 2021, which captured the B.1.1.7 (alpha; January 2021-June 2021) and B.1.617.2 (delta; June 2021**-**December 2021) variant waves but did not include data from the B.1.1.529 (omicron; December 2021**-**present) wave [39]. Due to the insufficient sample size of individuals vaccinated with non-mRNA vaccines, and the different mechanisms between the mRNA vaccine and adenovector vaccines (such as Johnson & Johnson) [40], we only investigated breakthrough infections in the fully mRNA-vaccinated individuals.

### Cohort Definition

Individuals over the age of 18 years who resided in NYC were included in this study. OMOP concepts related to vaccines were used to identify vaccinated individuals who received 2 doses of Pfizer/BNT162b2 or Moderna/mRNA-1273. To minimize potential bias resulting from missing vaccination records, vaccines records in our data warehouse were obtained from both CUIMC EHR data and the NYC vaccine registry. We required individuals to complete their 2-dose administration with a time interval of 20-23 days for Pfizer/BNT162b2 and 27-31 days for Moderna/mRNA-1273; individuals with 2 doses with 14 days of available follow-up after their second dose were considered fully vaccinated. Individuals who received doses from more than 1 manufacturer or only received 1 vaccine dose were excluded. We defined COVID-19-positive cases by using the OMOP measurement concepts and corresponding value concepts related to detect positive RNA using polymerase chain reaction (PCR). Individuals with at least 1 positive SARS-CoV-2 PCR test were flagged as COVID-19 positive. To balance the cofounding between positive cases and negative cases, we adopted a test-negative design—only individuals with at least 1 negative PCR test were included as COVID-19-negative cases. To reduce the potential false positives in the negative cohort, we additionally established stringent criteria to further exclude individuals with any evidence of a prior SARS-CoV-2 infection: (1) a positive SARS-CoV-2 PCR test, (2) a positive SARS-CoV-2 antibody test, or (3) a concept indicating SARS-CoV-2 infection. The details of OMOP concepts used for the cohort definition are available in Multimedia Appendix 1.

**Figure 1:**
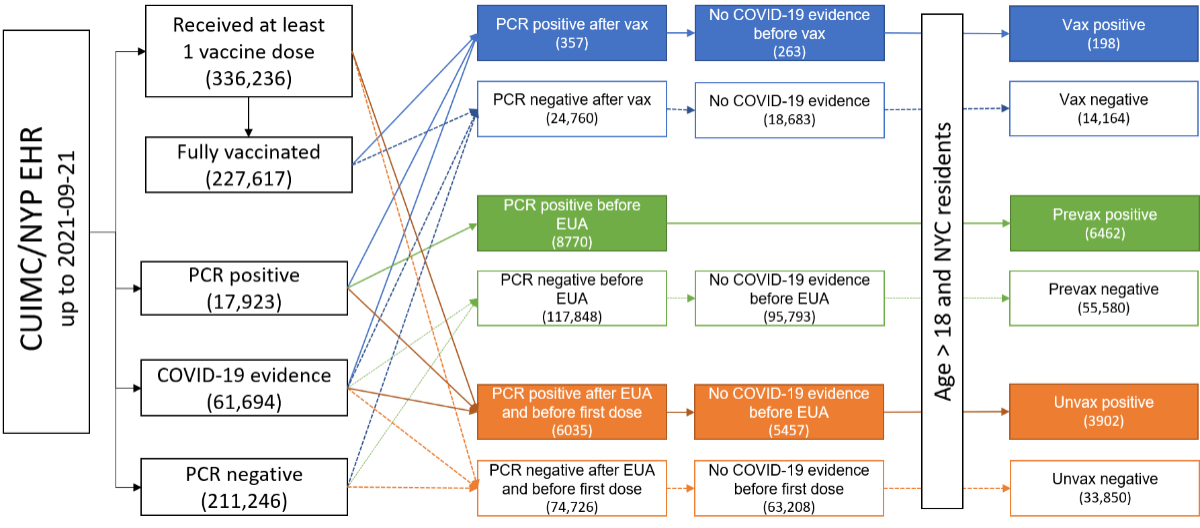
Cohort construction diagram and study overview. Vaccines records were obtained from both CUIMC EHR data and the NYC vaccine registry. Only fully vaccinated individuals with mRNA vaccines were included. Individuals with a positive SARS-CoV-2 PCR test, a positive SARS-CoV-2 antibody test, or a concept indicating a SARS-CoV-2 infection in the condition table were flagged as having evidence of SARS-CoV-2 infection. No COVID-19 evidence was required before entering the cohort for positive individuals and before exiting the cohort for negative individuals. Only age >18 years and NYC residents were included in this analysis. “Vax”: individuals 14 days after receiving their second doses were considered fully vaccinated; “EUA”: the date on which the first dose of the vaccine was administrated (ie, December 11, 2021); “first dose”: the date on which the individual was administrated their first (including Johnson & Johnson) vaccine dose (or the end of the study if a vaccine was not ever administrated). CUIMC/NYP: Columbia University Irving Medical Center/New York Presbyterian; EHR: electronic health record; EUA: Emergency Use Authorization; mRNA: messenger RNA; NYC: New York City; PCR: polymerase chain reaction.

Based on the vaccine and SARS-CoV-2 status, we then constructed 6 cohorts based on evidence of COVID-19 breakthrough infection (ie, positive or negative) and vaccination status (ie Vax, Prevax, and Unvax), as shown in Figure 1. For example, “Vax positive” is a collection of individuals who were vaccinated but later experienced breakthrough infections. The vaccination status was classified into “Vax” (those who were fully vaccinated), “Prevax” (those during a period when vaccines were unavailable), and “Unvax” (those who were not vaccinated during the period when vaccines were available). Individuals who were in the Prevax infection–negative cohort could also be in a Vax cohort later. Of note, if an individual receives a first dose for vaccination, that individual exits the Unvax cohort (and may later become part of a Vax cohort if fully vaccinated). More details about the cohort definitions can be found in the Results section.

### Feature Extraction

For each cohort, we extracted individuals' demographic data, including age, gender, ethnicity, and race. For the vaccinated cohorts, the vaccine brand and corresponding administration dates were also extracted. To approximate the available observation time, we extracted the total number of prior EHR visits and days of observation periods between clinical encounters for each individual. We extracted all previous condition and drug concepts from the *condition_era* and *drug_era* tables. To avoid extracting condition/drug concepts potentially caused by the SARS-CoV-2 infection itself, we added a 90-day washout period (ie, ignore all the concepts within the 90-day window prior to the PCR test regardless of its result). To identify individuals who might have compromised immune systems, we compiled a list of conditions and drugs, including active solid tumors and hematologic malignancies (within 2 years), solid-organ or hematopoietic stem cell transplant, primary immunodeficiencies, HIV infection, immunosuppressive therapies (eg, cancer chemotherapeutic agents, certain biologic agents, rituximab), and CKD [41]; see Multimedia Appendices 2 and 3. Individuals could fall into multiple immunocompromised subgroups. To adjust for the caseload in NYC, a 7-day rolling average of cases was applied [42].

### Identifying Risk Factors Associated With Breakthrough Infections

We compared the Vax-positive and Vax-negative cohorts to identify potential risk factors associated with breakthrough infections (Multimedia Appendix 4). The entry date was defined as the fully vaccinated date, and individuals were then followed until the first positive PCR date (or the end of the study for Vax-negative individuals). For each risk factor (eg, vaccine brand, demographics, immunocompromised status), a univariate Poisson regression was fit to assess the incidence rate ratio (IRR; ie, breakthrough per 1000 person-days) against the reference status. To minimize potential bias resulting from daily caseload, viral mutations, and EHR data quality, the Poisson regression was adjusted for (1) the total number of observation days in the EHR before the entry date, (2) the total number of visits in the EHR before the entry date, and (3) the calendar month of the PCR test date. We further applied a non-hypothesis-driven approach to uniformly evaluate the risk effect for each historical condition and drug by fitting a univariate Poisson regression with similar adjustment. Condition and drug concepts significantly associated with the breakthrough infections were identified as a <.05 Bonferroni-adjusted *P* value [43].

### Evaluation of Vaccine Effectiveness in Fully Vaccinated Individuals in Preventing Infection by Comparing Vaccinated Individuals With Pre- and Unvaccinated Individuals

For the Vax cohorts, the entry date was defined similarly to the entry date for the risk factor analysis. For the Unvax cohorts, the entry date was defined as January 18, 2021 (14 days after the first individual received their second dose at the CUIMC/NYP), and individuals were then followed up until the first positive PCR test (latest for negative individuals) or the date when they received their first dose, whichever came first (Multimedia Appendix 5). We 1:1-matched vaccinated individuals to unvaccinated individuals using a nearest-neighbor search based on (1) observation days, (2) visit count, (3) calendar week of the PCR test (earliest positive PCR or latest negative PCR), (4) demographics (eg, gender, age, race, ethnicity), and (5) immunocompromised status (binary). The IRRs for the vaccine were estimated via Poisson regressions.

As shown in Multimedia Appendix 6, we further identified 1:1-matched individuals in the Prevax cohort based on the same covariates, except for the calendar week of the PCR test, which was replaced by the 7-day rolling average of cases in NYC at the PCR testing date. Given the difficulty in identifying an appropriate entry date for the prevaccinated cohort, we applied a case-control design to calculate the odds ratio (OR) of contracting COVID-19 infection between the Vax cohort and the Prevax cohort using logistic regressions.

## Results

### Cohort Characteristics

Table 1 provides baseline characteristics of the 6 cohorts (note: some individuals are in multiple cohorts at different times). For the Vax-positive (ie, breakthrough) cohort, the median age was 60 years (IQR 40.7-75.4). Of 198 individuals in the Vax-positive cohort, 156 (78.8%) received Pfizer/BNT162b2, while 42 (21.2%) received Moderna/mRNA-1273. In addition, 65 (45.5%) had underlying immunocompromised conditions, and 120 (60.6%) of the patients with breakthrough infections were hospitalized. In general, PCR-positive individuals had a higher number of prior visits and observational days compared to unvaccinated individuals. For later analyses, we used a matching strategy to balance the covariates between the cohorts.

The overall estimated breakthrough infection rate was 0.16 (95% CI 0.14-0.18). Table 2 summarizes risk factors associated with breakthrough infections. We found a significantly higher incidence rate in vaccinated males than in females (IRR=1.47, 95% CI 1.11-1.94). We did not find any significant change in the incidence rate associated with other demographics, though Black individuals are likely to have a higher incidence rate and Asian individuals are likely to have a lower incidence rate. However, given the large portion of unknown race/ethnicity in the EHRs, our study was unable to estimate this association with meaningful accuracy. There was a significantly higher rate of breakthrough infections among those vaccinated with Pfizer/BNT162b2 compared to Moderna/mRNA-1273 (adjusted IRR=1.66, 95% CI 1.17-2.35). An immunocompromised state was significantly associated with a higher incidence rate among the vaccinated (adjusted IRR=1.48, 95% CI 1.09-2.00). Those with primary immunodeficiency, a history of organ transplant, an active tumor, and use of immunosuppressant medications were at the highest risk.

For the underlying conditions and drug usage analysis, a total of 1359 and 536 unique candidate conditions and drugs were available for investigation, respectively. Concepts needed a minimum of 100 individuals to be considered. Table 3 summarizes the top 10 breakthrough infection-associated condition and drug concepts. In addition to previously known conditions and drugs related to immunocompromised status (eg, immunodeficiency disorder, valganciclovir), we found that prior conditions and drugs related to pulmonary disease (eg, postinflammatory pulmonary fibrosis, albuterol) were also among those significantly associated with an increased breakthrough infection rate. The full list of associated conditions and drug concepts is provided in Multimedia Appendices 2 and 3.

We analyzed the protective effect of vaccination in the Vax cohort using 2 matched Prevax and Unvax cohorts. When comparing the Vax cohort with the Prevax cohort, the risk of COVID-19 infection in vaccinated individuals was significantly lower (adjusted OR 0.12, 95% CI 0.10-0.13), which was also the case when stratifying by age, gender, and immunocompromised status (Table 4). Similarly, we found a significant reduction in the incidence rate (adjusted IRR=0.42, 95% CI 0.36-0.49) when comparing the Vax cohort with the Unvax cohort (Table 5); similar observations were found across age, gender, and immunocompromised status subgroups.

**Table 1:** Baseline characteristics of the individuals in 6 cohorts (prematched).

Characteristics			Vax cohort				Unvax cohort				Prevax cohort		
			Vax positive^a^ (N=198)		Vax negative^b^ (N=14,164)	Unvax positive^c^ (N=3902)			Unvax negative^d^ (N=33,850)		Prevax positive^e^ (N=6462)		Prevax negative^f^ (N=55,580)
Entry date			Full vaccinated date		Full vaccinated date	January 18, 2021			January 18, 2021		January 1, 2020		January 1, 2020
End date			September 21, 2021		End of the study	Vaccination date or September 21, 2021			Vaccination date or September 21, 2021		December 10, 2020		December 10, 2020
Previous visit counts, mean (SD)			80 (124.75)		65.7 (121.91)	64 (121.15)			44.4 (91.4)		70.6 (127.86)		45.2 (95.1)
Observational days, mean (SD)			5470 (3909.61)		5425.2 (3843.99)	5940.6 (4045.09)			4999.3 (3799.28)		5942.1 (3978.71)		4932.6 (3672.63)
**Age (years), n (%)**													
	18-39	53 (26.8)		2995 (21.1)		1249 (32)		13,151 (38.9)		1401 (21.7)		19,074 (34.3)	
	40-59	42 (21.2)		3611 (25.5)		1167 (29.9)		10,363 (30.6)		1760 (27.2)		15,454 (27.8)	
	60-79	71 (35.9)		5547 (39.2)		1078 (27.6)		7836 (23.1)		2298 (35.6)		15,782 (28.4)	
	>=80	32 (16.2)		2011 (14.2)		408 (10.5)		2500 (7.4)		1003 (15.5)		5270 (9.5)	
**Gender, n (%)**													
	Female	110 (55.6)		9010 (63.6)		2199 (56.4)		21,065 (62.2)		3293 (51)		34,563 (62.2)	
	Male	88 (44.4)		5153 (36.4)		1702 (43.6)		12,765 (37.7)		3168 (49)		21,009 (37.8)	
	Unknown/other	N/A^g^		1 (0)		1 (0)		20 (0.1)		1 (0)		8 (0)	
**Race, n (%)**													
	Asian	3 (1.5)		545 (3.8)		73 (1.9)		804 (2.4)		132 (2)		2021 (3.6)	
	Black	30 (15.2)		1851 (13.1)		831 (21.3)		7046 (20.8)		1231 (19)		9218 (16.6)	
	White	88 (44.4)		6325 (44.7)		887 (22.7)		9740 (28.8)		1779 (27.5)		20,816 (37.5)	
	Unknown/other	77 (38.9)		5443 (38.4)		2111 (54.1)		16,260 (48)		3320 (51.4)		23,525 (42.3)	
**Ethnicity, n (%)**													
	Hispanic or Latino	58 (29.3)		3932 (27.8)		1840 (47.2)		12,081 (35.7)		2823 (43.7)		15,018 (27)	
	Not Hispanic or Latino	101 (51)		7571 (53.5)		1339 (34.3)		14,512 (42.9)		2224 (34.4)		27,194 (48.9)	
	Unknown/other	39 (19.7)		2661 (18.8)		723 (18.5)		7257 (21.4)		1415 (21.9)		13368 (24.1)	
**Vaccine brand, n (%)**													
	Moderna/mRNA^h^-1273	42 (21.2)		4626 (32.7)		N/A		N/A		N/A		N/A	
	Pfizer/BNT162b2	156 (78.8)		9538 (67.3)		N/A		N/A		N/A		N/A	
**Immunocompromised^i^, n (%)**													
	Solid tumor	46 (23.2)		2354 (16.6)		274 (7)		2826 (8.3)		629 (9.7)		6702 (12.1)	
	CKD^j^	28 (14.1)		1486 (10.5)		364 (9.3)		2124 (6.3)		910 (14.1)		4098 (7.4)	
	HIV	9 (4.5)		478 (3.4)		114 (2.9)		982 (2.9)		190 (2.9)		1603 (2.9)	
	On immunosuppressive therapy	13 (6.6)		362 (2.6)		74 (1.9)		616 (1.8)		156 (2.4)		1248 (2.2)	
	Immunodeficiency disorders	49 (24.7)		2545 (18)		370 (9.5)		3124 (9.2)		759 (11.7)		6660 (12)	
	Organ transplant	10 (5.1)		366 (2.6)		108 (2.8)		610 (1.8)		244 (3.8)		1288 (2.3)	
	None	108 (54.5)		9031 (63.8)		3072 (78.7)		26,835 (79.3)		4641 (71.8)		41,150 (74)	

^a^Individuals with a positive PCR^k^ test after full vaccination and without evidence of SARS-CoV-2 infection before full vaccination.

^b^Individuals with a negative PCR test after full vaccination and without evidence of SARS-CoV-2 infection at any time in their records.

^c^Individuals with a positive PCR test after the entry date and before administration of a first vaccination dose (if ever administrated), while having no evidence of SARS-CoV-2 infection before the entry date.

^d^Individuals with a negative PCR test after the entry date and before administration of a first vaccination dose (if ever administrated), while having no evidence of SARS-CoV-2 infection before the entry date.

^e^Individuals with a positive PCR test before the vaccination period.

^f^Individuals with a negative PCR test and without any evidence of SARS-CoV-2 infection before the vaccination period.

^g^N/A: not applicable.

^h^mRNA: messenger RNA.

^i^These are not mutually exclusive (except for the “None” category).

^j^CKD: chronic kidney disease.

^k^PCR: polymerase chain reaction.

**Table 2:** Risk factors associated with the breakthrough case rate in the CUIMC/NYP^a^.

Risk factors		Infection rate (95% CI) per 1000 person-days	IRR^b^ (95% CI)^c^	*P* value	Adjusted IRR (95% CI)^d^	*P* value adjusted
Overall		0.16 (0.14-0.18)	N/A^e^	N/A	N/A	N/A
**Age (years)**						
	18-39	0.19 (0.15-0.25)	Reference	Reference	N/A	N/A
	40-59	0.14 (0.10-0.19)	0.77 (0.51-1.17)	.22	N/A	N/A
	60-79	0.15 (0.11-0.19)	0.98 (0.66-1.47)	.93	N/A	N/A
	>=80	0.16 (0.11-0.23)	1.16 (0.70-1.91)	.56	N/A	N/A
**Gender**						
	Female	0.14 (0.11-0.17)	Reference	Reference	N/A	N/A
	Male	0.19 (0.16-0.24)	1.47 (1.11-1.94)	.01	N/A	N/A
**Race**						
	Asian	0.06 (0.01-0.18)	Reference	Reference	N/A	N/A
	Black	0.19 (0.13-0.27)	3.25 (0.99-10.70)	.05	N/A	N/A
	White	0.15 (0.12-0.19)	2.90 (0.91-9.19)	.071	N/A	N/A
	Unknown/other	0.17 (0.13-0.21)	2.88 (0.91-9.18)	.073	N/A	N/A
**Ethnicity**						
	Hispanic or Latino	0.18 (0.13-0.23)	Reference	Reference	N/A	N/A
	Not Hispanic or Latino	0.15 (0.12-0.18)	0.85 (0.60-1.21)	.37	N/A	N/A
	Unknown/other	0.17 (0.12-0.23)	0.91 (0.60-1.40)	.68	N/A	N/A
**Vaccine brand**						
	Moderna/mRNA^f^-1273	0.10 (0.07-0.14)	Reference	Reference	Reference	Reference
	Pfizer/BNT162b2	0.19 (0.16-0.22)	1.65 (1.17-2.33)	.005	1.66 (1.17-2.35)^g^	.004
**Immune system**						
	Not immunocompromised	0.14 (0.11-0.17)	Reference	Reference	Reference	Reference
	Is immunocompromised	0.19 (0.15-0.24)	1.49 (1.10-2.00)	.009	1.48 (1.09-2.00)	.011
	Active tumor	0.22 (0.16-0.29)	1.57 (1.11-2.21)	.01	1.56 (1.10-2.2)	.012
	CKD^h^	0.2 (0.13-0.29)	1.35 (0.89-2.07)	.16	1.33 (0.86-2.06)	.19
	HIV	0.21 (0.10-0.40)	1.24 (0.63-2.44)	.54	1.25 (0.63-2.47)	.52
	On immunosuppressed therapy	0.21 (0.16-0.28)	1.46 (1.03-2.05)	.03	1.45 (1.03-2.04)	.03
	Primary immunodeficiency	0.4 (0.21-0.68)	2.55 (1.41-4.60)	.002	2.53 (1.40-4.58)	.002
	Organ transplant	0.31 (0.15-0.57)	1.9 (0.98-3.71)	.059	1.9 (0.98-3.71)	.058

^a^CUIMC/NYP: Columbia University Irving Medical Center/New York Presbyterian.

^b^IRR: incidence rate ratio.

^c^Adjusted for number of visits, days of previous observation, and calendar month of the PCR^i^ test result.

^d^Adjusted for number of visits, days of previous observation, calendar month of the PCR test result, and age at the last vaccine dose.

^e^N/A: not applicable.

^f^mRNA: messenger RNA.

^g^Adjusted for number of visits, days of previous observation, calendar month of the PCR test result, age at the last vaccine dose, and whether the immune system is compromised.

^h^CKD: chronic kidney disease.

^i^PCR: polymerase chain reaction.

**Table 3:** Top 10 (ranked by *P* value) condition and drug concepts associated with breakthrough cases in the Vax cohort in the CUIMC/NYP^a^.

OMOP^b^ concept ID^c^		IRR^d^ (95% CI)^e^	*P* value	Condition name
**Conditions**				
	315831	4.07 (2.07-7.99)	<.001	Chronic pulmonary heart disease
	4228361	2.60 (1.56-4.33)	<.001	Asteatosis cutis
	433740	3.62 (1.81-7.22)	<.001	Immunodeficiency disorder
	253797	3.34 (1.69-6.59)	<.001	Postinflammatory pulmonary fibrosis
	4177206	3.84 (1.78-8.28)	.001	Tubulointerstitial nephritis
	378419	3.50 (1.68-7.28)	.001	Alzheimer disease
	257315	2.97 (1.05-5.87)	.002	Bacterial pneumonia
	4170770	2.45 (1.39-4.32)	.002	Epidermoid cyst
	443729	2.78 (1.45-5.36)	.002	Peripheral circulatory disorder due to type 2 diabetes mellitus
	44782747	3.62 (1.58-8.27)	.002	Acute deep venous thrombosis of femoral vein
**Drugs**				
	1703063	4.33 (1.92-9.76)	<.001	Valganciclovir
	715997	2.91 (1.50-5.65)	.002	Donepezil
	1325608	3.62 (1.54-8.49)	.003	Pegfilgrastim
	19008339	3.27 (1.42-7.53)	.005	Vitamin A
	1317640	3.18 (1.40-7.24)	.006	Telmisartan
	1154343	1.56 (1.13-2.15)	.007	Albuterol
	40239216	3.01 (1.32-6.86)	.009	Linagliptin
	1341927	2.21 (1.21-4.02)	.01	Enalapril
	1149196	1.93 (1.17-3.17)	.01	Cetirizine
	19003999	2.77 (1.27-6.04)	.01	Mycophenolate mofetil

^a^CUIMC/NYP: Columbia University Irving Medical Center/New York Presbyterian.

^b^OMOP: Observational Medical Outcomes Partnership.

^c^Only concepts that occurred in more than 100 individuals were included in this analysis.

^d^IRR: incidence rate ratio.

^e^Poisson regression was fitted for each variable with adjustment for age, number of visits, and observational days.

**Table 4:** Vaccine effectiveness against SARS-CoV-2 infection comparing the Vax cohort with a matched Prevax cohort before December 11, 2020.

Characteristics			Prevax/Vax^a^, n (%)		Prevalence (Precax/Vax), n (%)		OR^b^ (95% CI)^c^	Adjusted OR (95% CI)^d^
Overall			14,362 (100)/14,362 (100)		1556 (100)/198 (100)		0.12 (0.10-0.13)	0.12 (0.10-0.14)
**Age (years)**								
	18-39	2997 (20.9)/3048 (21.2)		206 (13.2)/53 (26.8)		0.24 (0.18-0.32)		0.25 (0.18-0.34)
	40-59	3788 (26.4)/3653 (25.5)		338 (21.7)/42 (21.2)		0.12 (0.09-0.16)		0.12 (0.09-0.17)
	60-79	5218 (36.3)/5618 (39.1)		636 (40.9)/71 (35.8)		0.09 (0.07-0.12)		0.09 (0.07-0.12)
	>=80	2359 (16.4)/2043 (14.2)		376 (24.2)/32 (16.2)		0.08 (0.06-0.12)		0.08 (0.06-0.12)
**Gender**								
	Male	5142 (35.8)/5241 (36.5)		702 (45.1)/88 (44.4)		0.11(0.09-0.14)		0.11 (0.09-0.14)
	Female	9220 (64.2)/9120 (63.5)		854 (54.9)/110 (55.6)		0.12 (0.10-0.15)		0.12 (0.10-0.15)
**Is immunocompromised**								
	True	5287 (36.8)/5223 (36.4)		642 (41.3)/90 (45.5)		0.13 (0.10-0.16)		0.13 (0.10-0.16)
	False	9075 (63.2)/9139 (63.6)		914 (58.7)/108 (54.5)		0.11 (0.09-0.13)		0.11 (0.09-0.13)

^a^Each cohort contained 14,362 individuals in total because of 1:1 matching; matching was based on previous visit counts, observational days, demographics, underlying immune conditions, and the NYC^e^ 7-day rolling average of COVID-19 cases on the PCR^f^ test date.

^b^OR: odds ratio.

^c^OR obtained by fitting a univariate logistic regression between the Vax cohort and a matched Prevax cohort.

^d^OR obtained by fitting a logistics regression adjusted for the previous number of visits and observational days.

^e^NYC: New York City.

^f^PCR: polymerase chain reaction.

**Table 5:** Vaccine effectiveness against SARS-CoV-2 infection comparing the Vax cohort with a matched Unvax cohort after June 18, 2021.

Characteristics			Unvax/Vax^a^, n (%)		Incidence rate/1000 person-days (Unvax/Vax)		IRR^b^ (95% CI)^c^	Adjusted IRR (95% CI)^d^	
Overall			14,362 (100)/14,362 (100)		0.37/0.16		0.42 (0.36-0.49)	0.41 (0.35-0.48)	
**Age (years)**									
	18-39	3748 (26.1)/3048 (21.2)		0.32/0.2		0.63 (0.46-0.85)		0.64 (0.47-0.87)	
	40-59	4216 (29.4)/3653 (25.4)		0.37/0.14		0.38 (0.28-0.53)		0.38 (0.27-0.52)	
	60-79	4548 (31.7)/5618 (39.1)		0.39/0.15		0.37 (0.28-0.48)		0.35 (0.27-0.46)	
	>=80	1850 (12.9)/2043 (14.2)		0.47/0.16		0.34 (0.23-0.50)		0.31 (0.21-0.46)	
**Gender**									
	Male	5272 (36.7)/5241 (36.5)		0.4/0.19		0.49 (0.39-0.62)		0.48 (0.38-0.61)	
	Female	9089 (63.3)/9120 (63.5)		0.36/0.14		0.38 (0.31-0.47)		0.37 (0.30-0.45)	
**Is immunocompromised**									
	True	4079 (28.4)/5223 (36.4)		0.41/0.19		0.47 (0.37-0.59)		0.43 (0.34-0.55)	
	False	10,283 (71.6)/9139 (63.6)		0.36/0.14		0.38 (0.31-0.47)		0.38 (0.31-0.46)	

^a^Each cohort contained 14,362 individuals in total because of 1:1 matching; matching was based on previous visit counts, observational days, demographics, underlying immune conditions, and the NYC^e^ 7-day rolling average of COVID-19 cases on the PCR^f^ test date.

^b^IRR: incidence rate ratio.

^c^IRR obtained by fitting a univariate Poisson regression between the Vax cohort and a matched Unvax cohort.

^d^IRR obtained by fitting a Poisson regression adjusted for the previous number of visits and observational days.

^e^NYC: New York City.

^f^PCR: polymerase chain reaction.

## Discussion

### Principal Findings

By comparing the breakthrough cohort (ie, Vax positive) against the no-breakthrough cohort (ie, Vax negative), we found a number of medical commodities were associated with an increased risk of breakthrough infection. First, we found that immunosuppressive therapy is associated with higher rates of breakthrough infection. Individuals with active tumors also had higher rates of breakthrough infection, suggesting that the effects of active malignancy or chemotherapy lead to a reduced immune response. There was a statistically not significant increase in individuals with a history of tumors, suggesting that individuals whose cancers are in remission are more similar to the average population in terms of immune response. Our findings are in line with prior studies of solid organ transplant recipients who have shown weaker immune responses in patients who are immunosuppressed and undergo vaccination against COVID-19 [44,45]. For example, valganciclovir is a drug used commonly to prevent cytomegalovirus disease after solid organ transplantation [46], and we found it was significantly associated with the increased risk of breakthrough infection, indicating individuals who underwent solid organ transplant were among those at high risk of breakthrough infections. We also observed an increased risk of infection in individuals with prior lung infection. A potential explanation is the microbiome changes within the lung that play a key role in the initiation and progression of COVID-19 [47,48]. In addition, studies have shown that patients with COVID-19 and preexisting interstitial lung disease (ILD) had a poorer prognosis [49,50], which highlights the importance of staying vigilant and continued use of personal protective and social measures, even with vaccination among those individuals. Furthermore, in individuals with Alzheimer disease who were vaccinated, there was an increased risk of infection, which might be due to their frailty and medical vulnerability, and nonadherence to infection control measures, such as physical distancing [51]. This is also confirmed by the finding that donepezil is a high-risk factor, which is used to treat confusion (dementia) related to Alzheimer disease [52]. We did not find a significantly increased risk of breakthrough infection in individuals with CKD. An ongoing study (the Renal Patients COVID-19 Vaccination Immune Response [RECOVAC-IR] study) aims to provide further guidance regarding the efficacy of vaccines in patients with CKD or whether other measures, such as booster vaccinations, are required [53].

Although our findings reaffirmed the high protection of mRNA vaccines against COVID-19 infection, we found that Moderna/mRNA-1273 had an overall higher effectiveness in preventing SARS-CoV-2 infections. A previous high-quality prospective study [54] involving 3975 individuals observed through April 2021 demonstrated similar vaccine effectiveness between the mRNA vaccines, but it was underpowered and did not perform statistical analysis. A more recent Mayo Clinic study of data collected through July 2021 [55] was consistent with our findings despite differences in cohort definitions and geography. Another recent study comparing the SARS-CoV-2 antibody response following vaccination similarly found higher antibody titers in participants vaccinated with mRNA-1273 compared with those vaccinated with Pfizer/BNT162b258. A more recent meta-analysis study of data collected through September 2021 showed that the estimated long-term vaccine effectiveness for COVID-19 hospitalization was 85.4% (95% CI 84.8%-86.0%) with the Pfizer/BNT162b2 vaccine and 89.8% (95% CI 89.2%-90.4%) with Moderna/mRNA-1273. Additional studies should be considered to provide further guidance on effectiveness differences between vaccine brands and booster shot prioritization. Although individuals with immunosuppressed disorders are at higher risk of developing breakthrough infection, the adjusted IRR in immunocompromised individuals is 0.43 (95% CI 0.34-0.55), supporting the conclusion that vaccination can still greatly reduce the infection rate among this subgroup [56]. Our study supports the current policies recommending that immunocompromised individuals receive booster doses [57].

It is important to provide constant public health surveillance of vaccine protection. By leveraging EHR data from various health systems, we can provide more robust and generalizable evidence of vaccine effectiveness. Unfortunately, it is not always easy to aggregate the medical data from multiple institutions due to Health Insurance Portability and Accountability Act of 1996 (HIPPA) constrains. Therefore, we developed an entirely CDM-based analysis pipeline, making it easily transferable to dozens of other health care databases compatible with it [58]. We have provided our OMOP-compatible analysis pipeline on the GitHub repository [59]. Other institutions that have implemented their OMOP instance can download the code and easily replicate the analysis using their own institution’s OMOP instance and share the evidence in a timely manner.

### Limitations

Given the high level of missingness typically found in EHR data, it is challenging to estimate the absolute incidence rate of breakthrough infections. In our study, the incidence rate among the vaccinated cohort was estimated to be 0.16 per 1000 person-days. This potentially overestimates the incidence rate (particularly in comparison to 0.031 in Israel’s national surveillance data [60], ~0.01 in the original Pfizer/BNT162b2 and Moderna/mRNA-1273 trials [1,2]) because we imposed a criterion to only include those who have at least 1 PCR test available, which is also called test-negative design [61]. If we remove this requirement, the incidence rate among the vaccinated cohort becomes ~0.007 per 1000 person-days. However, this is an underestimation of the true rate because some of the SARS-CoV-2-infected individuals might have been tested elsewhere or not at all. In addition, despite adopting a test-negative design, we were still unable to confirm whether negative cases were truly negative (eg, tested positive elsewhere). Similarly, some patients may be incorrectly labeled as unvaccinated if their vaccinations took place outside of NYC or the NYP health system, which could lower effectiveness estimations. However, our main focus in this study was to identify the risk subgroups at increased risk of breakthrough infections, and a cofounding-aligned comparative design can achieve this goal by matching the patient’s demographics and their tendency in seeking health care in our medical center.

Another limitation of this study is we could not stratify breakthrough infections by variant type due to limitations in testing data. Our study used the EHR data collected through September 2021, which covers periods where the B.1.1.7 (alpha) and B.1.617.2 (delta) variants were prevalent. Our findings cannot be generalized to other newly emerged variants of concerns, including B.1.1.529 (omicron) for which the existing mRNA vaccines may have differing effectiveness. However, this is not unique to our study, as the pandemic has often evolved faster than high-quality analyses can be performed. Even a recent systematic review of the efficacy and effectiveness of the COVID-19 vaccines published in January 2022 included only papers before April 2021 (the data collected in those papers can be from even earlier). The conflict between the speed of scientific publication and the rapid evolution of the pandemic remains a significant challenge for the overall research community.

Finally, the CUIMC/NYP is an academic medical center in NYC, which might not represent the general American population or other potential patient groups of interest. In particular, the overall population in our study is sicker than the general population, as evidenced by the high rate of comorbidities and older age of our patient cohort.

### Conclusion

We performed a retrospective analysis to investigate risk factors contributing to COVID-19 breakthrough infections among vaccinated individuals. We found those who are male, immunocompromised, or have preexisting pulmonary disease are at a higher risk of COVID-19 breakthrough infection. Although both vaccines are highly effective in preventing SARS-CoV-2 infection, Moderna/mRNA-1273 is associated with a lower risk of breakthrough infection than Pfizer/BNT162b2. Multiple medical institutions’ data are warranted to better link the PCR test results and vaccination information. Those with an OMOP instance of their data can reapply our analysis to check the robustness of our results [59].

## Appendix

Multimedia Appendix 1 The details of OMOP concepts used for cohort definition. OMOP: Observational Medical Outcomes Partnership.

Multimedia Appendix 2 The full list of breakthrough-associated condition concepts.

Multimedia Appendix 3 The full list of breakthrough-associated drug concepts.

Multimedia Appendix 4 Study design in identifying risk factors for breakthrough events by comparing PCR-positive cases and PCR-negative cases among vaccinated individuals. PCR: Polymerase Chain Reaction.

Multimedia Appendix 5 Study design in assessing vaccine effectiveness by comparing a vaccinated cohort and a matched unvaccinated cohort.

Multimedia Appendix 6 Study design in assessing vaccine effectiveness by comparing a vaccinated cohort and a matched prevaccinated cohort.

## Abbreviations

CDC: Centers for Disease Control and Prevention
CDM: Common Data Model
CKD: chronic kidney disease
CUIMC/NYP: Columbia University Irving Medical Center/New York Presbyterian
EHR: electronic health record
IRR: incidence rate ratio
mRNA: messenger RNA
NYC: New York City
OMOP: Observational Medical Outcomes Partnership
OR: odds ratio
PCR: polymerase chain reaction
