# Supplementary material for: Risk Factors Associated With SARS-CoV-2 Breakthrough Infections in Fully mRNA-Vaccinated Individuals: Retrospective Analysis
Source: JMIR Public Health Surveill. 2022 May 24;8(5):e35311. doi: 10.2196/35311 (PMC9132195; doi:10.2196/35311)
Supplement: Multimedia Appendix 4 [file publichealth_v8i5e35311_app4.pdf]

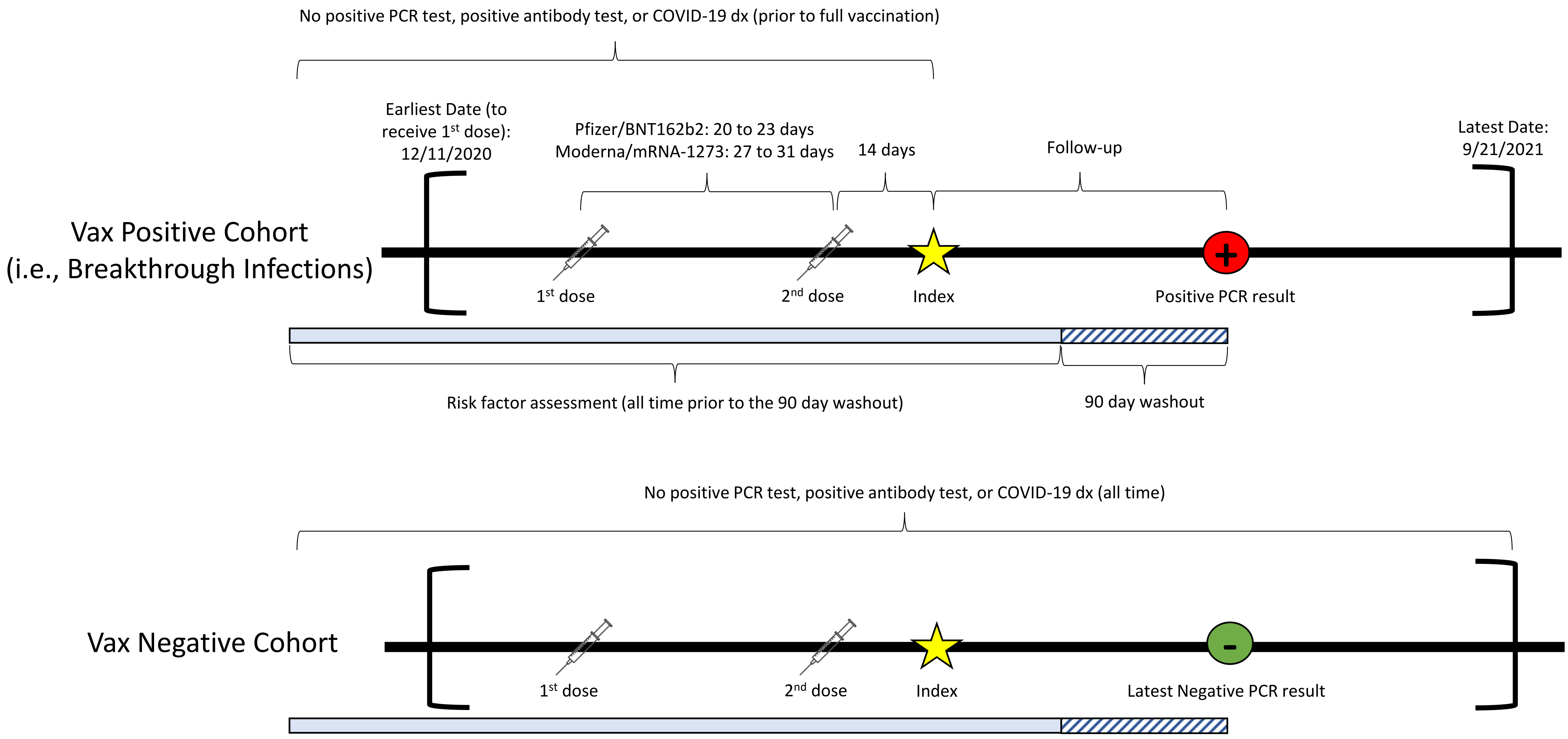
No positive PCR test, positive antibody test, or COVID-19 dx (prior to full vaccination)
Earliest Date (to receive 1st dose): 12/11/2020
Pfizer/BNT162b2: 20 to 23 days
Moderna/mRNA-1273: 27 to 31 days
14 days
Follow-up
Latest Date: 9/21/2021
Vax Positive Cohort (i.e., Breakthrough Infections)
1st dose
2nd dose
Index
Positive PCR result
Risk factor assessment (all time prior to the 90 day washout)
90 day washout
No positive PCR test, positive antibody test, or COVID-19 dx (all time)
Vax Negative Cohort
1st dose
2nd dose
Index
Latest Negative PCR result
