# Supplementary figures and images for: Risk Factors Associated With SARS-CoV-2 Breakthrough Infections in Fully mRNA-Vaccinated Individuals: Retrospective Analysis
Source: JMIR Public Health Surveill. 2022 May 24;8(5):e35311. doi: 10.2196/35311 (PMC9132195; doi:10.2196/35311)

## Un-Vax Cohort

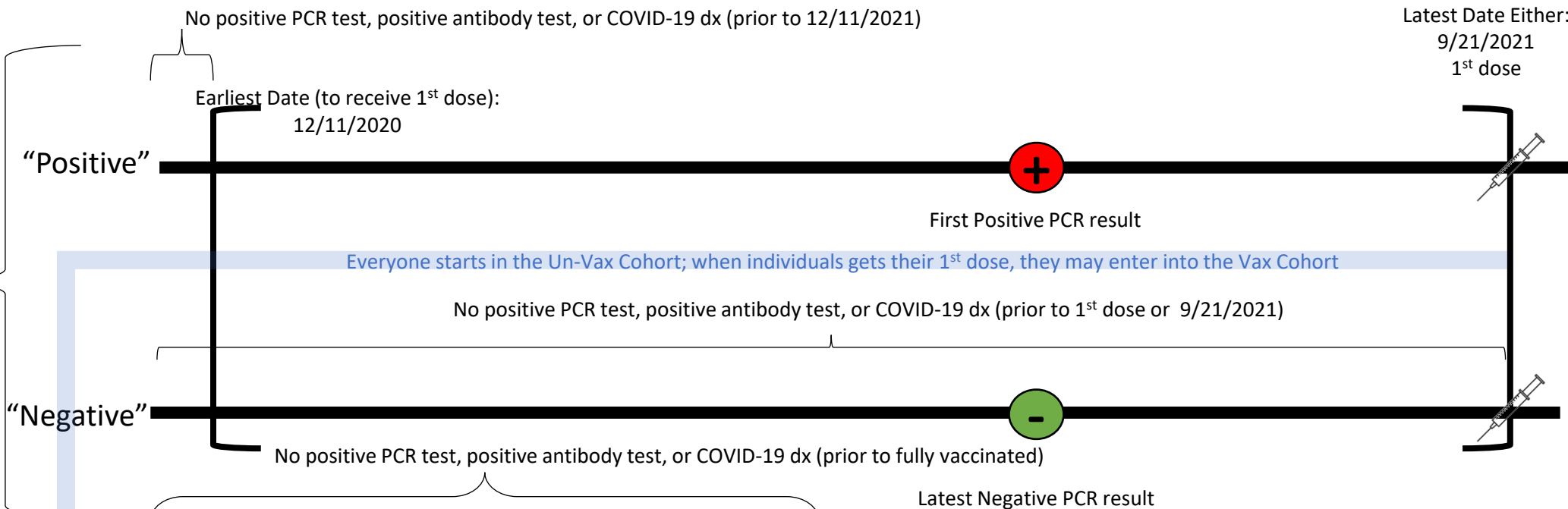

## Vax Cohort

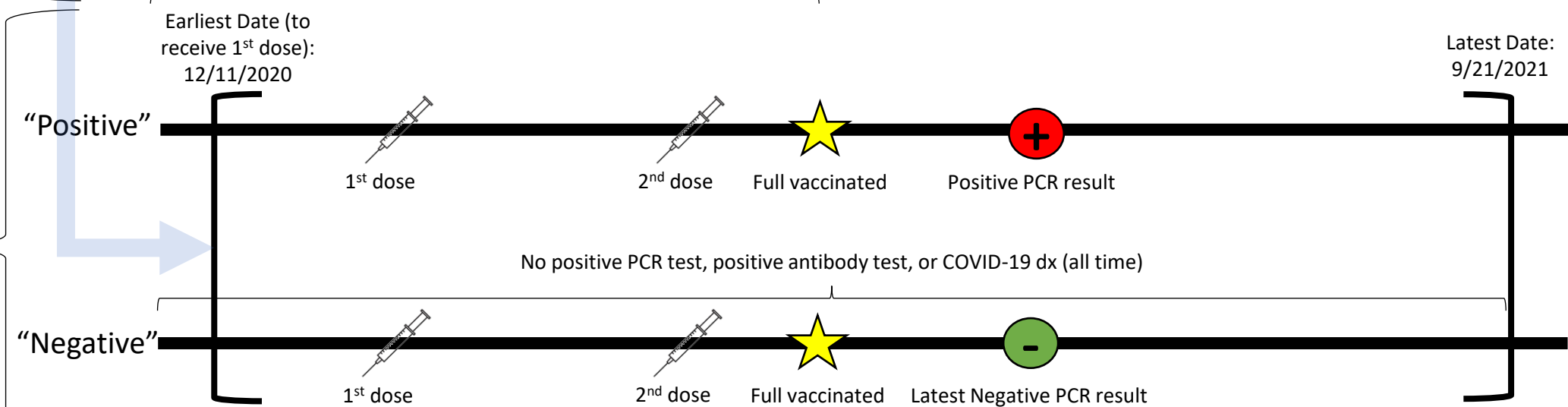

Supplement: Multimedia Appendix 5 [file publichealth_v8i5e35311_app5.pdf]

## Vax Cohort

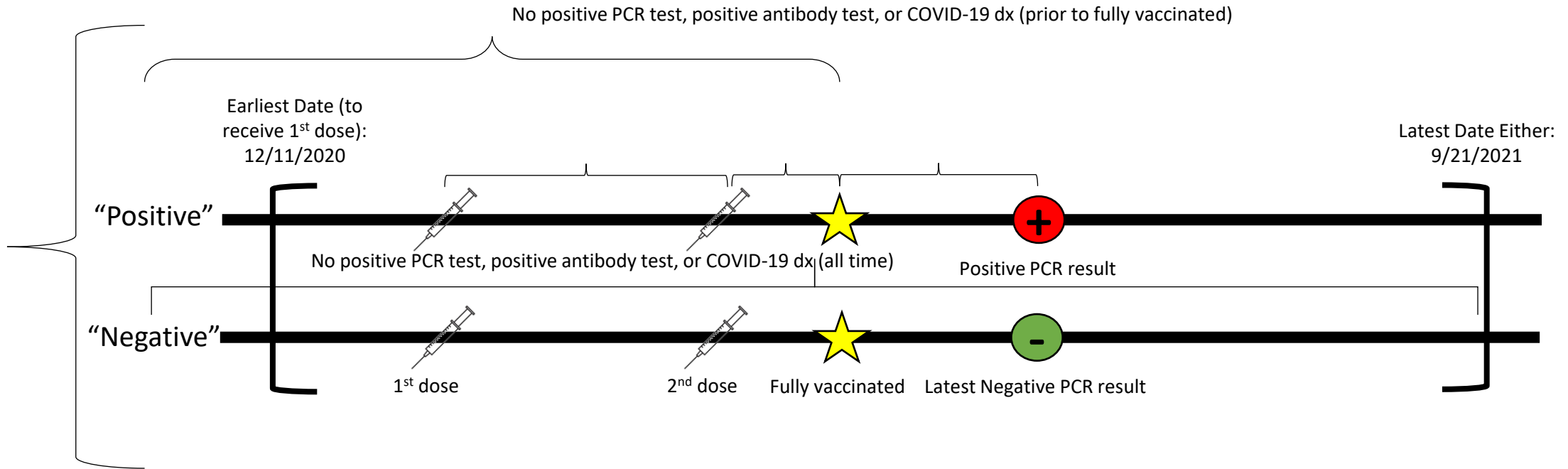

## Pre-Vax Cohort

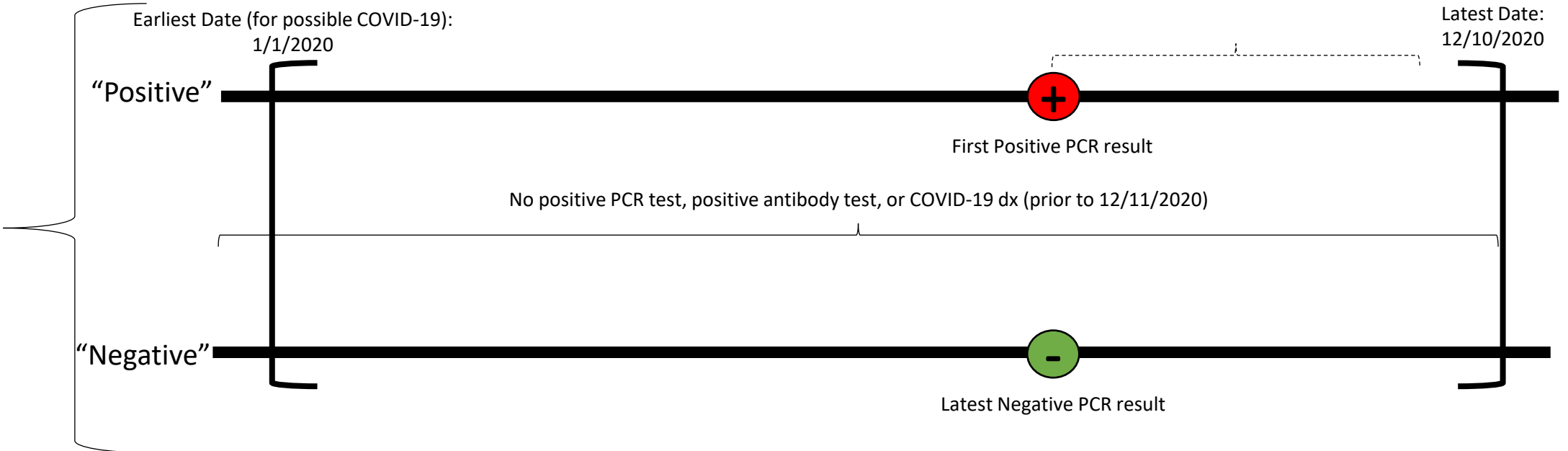

Supplement: Multimedia Appendix 6 [file publichealth_v8i5e35311_app6.pdf]
